# Differential thermostability and response to cystic fibrosis transmembrane conductance regulator potentiators of human and mouse F508del-CFTR

**DOI:** 10.1152/ajplung.00034.2019

**Published:** 2019-04-10

**Authors:** Samuel J. Bose, Marcel J. C. Bijvelds, Yiting Wang, Jia Liu, Zhiwei Cai, Alice G. M. Bot, Hugo R. de Jonge, David N. Sheppard

**Affiliations:** ^1^School of Physiology, Pharmacology and Neuroscience, University of Bristol, Bristol, United Kingdom; ^2^Department of Gastroenterology and Hepatology, Erasmus University Medical Center, Rotterdam, The Netherlands

**Keywords:** CFTR chloride ion channel, CFTR potentiation, cystic fibrosis, F508del-CFTR, ivacaftor (VX-770)

## Abstract

Cross-species comparative studies have highlighted differences between human and mouse cystic fibrosis transmembrane conductance regulator (CFTR), the epithelial Cl^−^ channel defective in cystic fibrosis (CF). Here, we compare the impact of the most common CF mutation F508del on the function of human and mouse CFTR heterologously expressed in mammalian cells and their response to CFTR modulators using the iodide efflux and patch-clamp techniques. Once delivered to the plasma membrane, human F508del-CFTR exhibited a severe gating defect characterized by infrequent channel openings and was thermally unstable, deactivating within minutes at 37°C. By contrast, the F508del mutation was without effect on the gating pattern of mouse CFTR, and channel activity demonstrated thermostability at 37°C. Strikingly, at all concentrations tested, the clinically approved CFTR potentiator ivacaftor was without effect on the mouse F508del-CFTR Cl^−^ channel. Moreover, eight CFTR potentiators, including ivacaftor, failed to generate CFTR-mediated iodide efflux from CHO cells expressing mouse F508del-CFTR. However, they all produced CFTR-mediated iodide efflux with human F508del-CFTR-expressing CHO cells, while fifteen CFTR correctors rescued the plasma membrane expression of both human and mouse F508del-CFTR. Interestingly, the CFTR potentiator genistein enhanced CFTR-mediated iodide efflux from CHO cells expressing either human or mouse F508del-CFTR, whereas it only potentiated human F508del-CFTR Cl^−^ channels in cell-free membrane patches, suggesting that its action on mouse F508del-CFTR is indirect. Thus, the F508del mutation has distinct effects on human and mouse CFTR Cl^−^ channels.

## INTRODUCTION

The most frequent cause of the life-limiting genetic disease cystic fibrosis (CF) is the F508del mutation in the cystic fibrosis transmembrane conductance regulator (CFTR) (23, 62, 63). CFTR is an ATP-binding cassette (ABC) transporter (ABCC7) (37), which functions as an ATP-gated anion channel regulated by cAMP-dependent phosphorylation (40). Located in the apical membrane of epithelia-lining ducts and tubes, it plays a key role in the regulation of transepithelial fluid and electrolyte movement (6, 68). Deletion of F508 causes a temperature-sensitive folding defect, which disrupts CFTR processing and intracellular transport to the plasma membrane (16, 26). But, in addition, the mutation destabilizes any CFTR protein that reaches the plasma membrane and interferes with channel gating, reducing greatly the frequency of channel opening (24, 48). To overcome these defects in F508del-CFTR, orally bioavailable small molecules termed CFTR correctors and potentiators have been developed (34, 43, 52). CFTR correctors repair misfolding of nucleotide-binding domain 1 (NBD1) and facilitate correct domain assembly, leading to the plasma membrane expression of F508del-CFTR protein (29, 53, 83). By contrast, CFTR potentiators increase the frequency and duration of channel openings to restore channel activity to F508del-CFTR (28, 41, 82). Additional beneficial actions of CFTR modulators include dampening inflammatory responses in CF airway epithelia (67) and restoring bacterial killing to CF macrophages (4).

To understand how the F508del mutation causes organ-level disease and assist the evaluation of innovative CF therapeutics, F508del-CFTR mouse models have been developed (19, 81, 95). Studies of these and other mouse models have highlighted differences in the pathophysiology of CF between humans and mice (for review, see Ref. 92). In part, these differences are explained by the distinct anatomy and physiology of humans and mice, which include variation in the expression of ion channels and transporters between the two species. For example, altered expression of the calcium-activated Cl^−^ channel TMEM16A might protect CF mice from pancreatic disease (18, 56), whereas the absence of the nongastric H^+^,K^+^-ATPase ATP12A might defend CF mice from lung infection (74). Interestingly, several lines of evidence suggest that differences in CFTR expression, structure, and function might also contribute to the distinct presentation of CF in humans and mice. First, the amino acid sequence of mouse CFTR differs noticeably from that of human CFTR (shared amino acid identity, 79%), exhibiting a higher degree of variation than that predicted by the phylogenetic tree (9, 79). Second, the F508del processing defect is less severe for mouse than for human CFTR (55). Third, there are striking differences in single-channel behavior between human and mouse CFTR (46, 72). Finally, mouse CFTR appears unresponsive to some but not all CFTR modulators (45, 64), while conflicting results have been obtained with the CFTR potentiator ivacaftor (20–22, 82, 96).

In this study, we investigated the impact of the F508del mutation on mouse CFTR. Using CHO cells heterologously expressing mouse F508del-CFTR and the patch-clamp and iodide efflux techniques, we studied its single-channel behavior, thermostability, and rescue by CFTR modulators. In marked contrast to the mutation’s severe impact on human CFTR, F508del was without effect on the gating behavior of mouse CFTR and its stability in cell-free membrane patches at 37°C. Similarly, most CFTR potentiators, including ivacaftor, failed to augment the activity of mouse F508del-CFTR, whereas CFTR correctors, including lumacaftor, rescued both human and mouse F508del-CFTR. Thus, subtle changes in protein structure influence the action of the F508del mutation on CFTR function and the effects of CFTR potentiators.

## METHODS

### 

#### Cells and cell culture.

**Fig. 1.**
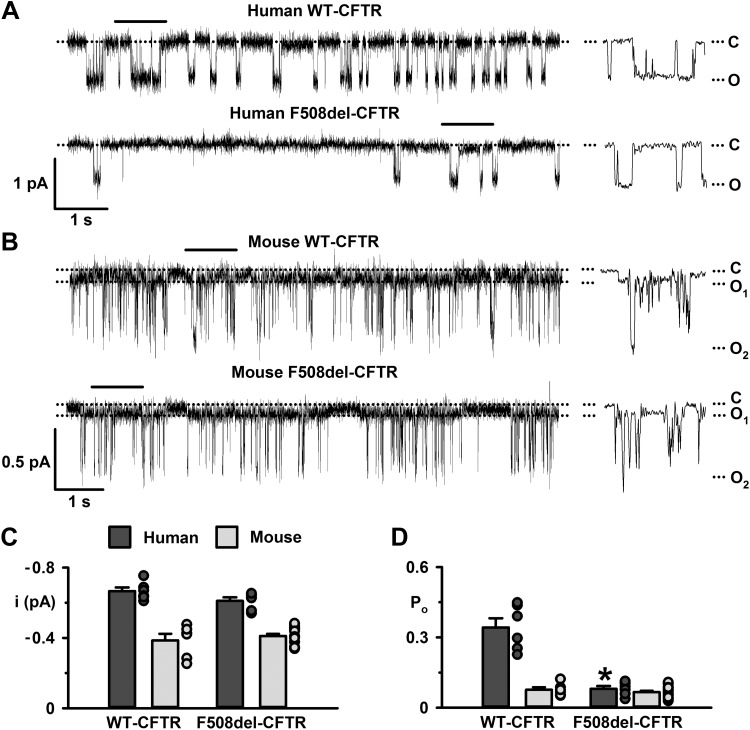
The single-channel behavior of human and mouse wild-type (WT) and F508del-cystic fibrosis transmembrane conductance regulator (CFTR). *A* and *B*: representative recordings of human and mouse wild-type and F508del-CFTR Cl^−^ channels in excised inside-out membrane patches from NIH-3T3 and Chinese hamster ovary (CHO) cells heterologously expressing CFTR variants. Prior to study, the plasma membrane expression of human and mouse F508del-CFTR was rescued by low-temperature incubation. The recordings were acquired at 37°C in the presence of ATP (1 mM) and PKA (75 nM) in the intracellular solution. The closed-channel state (C), the subconductance state of mouse CFTR (O_1_), and the full open state [human (O), mouse (O_2_)] are indicated by dotted lines. Traces on the *left* were filtered at 500 Hz, whereas the 1-s portions indicated by the bars shown on an expanded time scale to the *right* were filtered at 50 Hz. In this and subsequent figures with single-channel data, a large Cl^−^ concentration gradient was imposed across excised membrane patches ([Cl^−^]_int_, 147 mM; [Cl^−^]_ext_, 10 mM), and membrane voltage was clamped at –50 mV. *C* and *D*: summary single-channel current amplitude (*i*) and open probability (*P*_o_) data for the full open states of human and mouse CFTR determined from prolonged recordings (≥5 min) acquired from baby hamster kidney (BHK) and NIH-3T3 cells heterologously expressing human CFTR and CHO cells heterologously expressing mouse CFTR using the conditions described in *A* and *B* before channel deactivation (human F508del-CFTR). Dark gray and light gray circles represent individual values and columns means ± SE (human wild-type, *n* = 6; human F508del-CFTR, *n* = 6; mouse wild type, *n* = 6; mouse F508del-CFTR, *n* = 18); *P < 0.05 vs. human wild-type CFTR.

We used Chinese hamster ovary (CHO-K1) cells stably expressing human and mouse wild-type and F508del-CFTR (45). Because single-channel recording was not feasible with CHO cells expressing human F508del-CFTR (Bose SJ, Cai Z, and Sheppard DN, unpublished observations), we used NIH-3T3 cells and BHK cells stably expressing human wild-type and F508del-CFTR for patch-clamp studies (7, 30). Cells were generous gifts of J. R. Riordan (human CFTR-expressing CHO cells; University of North Carolina), B. J. Wainwright (mouse CFTR-expressing CHO cells; University of Queensland), M. D. Amaral (human CFTR-expressing BHK cells; University of Lisboa), and M. J. Welsh (human CFTR-expressing NIH 3T3 cells; University of Iowa). CHO cells were cultured in Ham’s F-12 nutrient medium supplemented with 10% fetal bovine serum (FBS), 100 U/ml penicillin, 100 μg/ml streptomycin, and either 200 μg/ml neomycin (mouse wild-type CFTR) or 220 μg/ml methotrexate (mouse F508del-CFTR). BHK cells were cultured as previously described (70), while NIH-3T3 cells were cultured in Dulbecco’s modified Eagle’s medium supplemented with 10% FBS, 100 U/ml penicillin, 100 μg/ml streptomycin, and 380 μg/ml G418 (F508del-CFTR-expressing NIH-3T3 cells only). All cells were cultured at 37°C in a humidified atmosphere containing 5% CO_2_. For patch-clamp experiments, cells were seeded onto glass coverslips and used within 7 days, with the media changed every 2 days. For ^125^I^−^ efflux experiments, cells were grown as monolayers to 70–80% confluence in tissue culture-treated six-well cell culture plates. To rescue the plasma membrane expression of F508del-CFTR, cells were incubated at 26–27°C for 24–96 h (26) or treated with lumacaftor (3 μM) for 24–48 h at 37°C (83). The single-channel behavior of human CFTR in excised membrane patches from different mammalian cell lines is equivalent [wild-type CFTR (15); F508del-CFTR: BHK cells, *i* = −0.72 ± 0.01 pA, *P*_o_ = 0.07 ± 0.01, *n* = 20; C127 cells, *i* = –0.78 ± 0.02 pA, *P*_o_ = 0.06 ± 0.01, *n* = 12 using the conditions described in Fig. 1]. Previous work (46, 55) also suggests that the single-channel properties of mouse CFTR are comparable using different mammalian cells.

#### Iodide efflux experiments.

Monolayers of CHO cells expressing human and mouse wild-type and F508del-CFTR were loaded with ^125^I^−^ for 2 h under a humidified atmosphere of O_2_/CO_2_ (19:1) at 37°C in 0.5 ml of isotonic medium containing (in mM) 130 NaCl, 5 KCl, 1.3 CaCl_2_, 1 MgCl_2_, 10 glucose, and 20 HEPES (pH 7.40) and 5 μCi/ml ^125^I^−^. Then, extracellular ^125^I^−^ was removed within 1 min by washing the cells three times with 3 ml of isotonic medium (without ^125^I^−^) at room temperature. Efflux of ^125^I^−^ was measured at 37°C by addition and consecutive removal of 1 ml of the isotonic medium without ^125^I^−^ at 1- to 2-min intervals. At the end of the experiment, residual ^125^I^−^ was determined by collecting the cells in 1 ml of 1 M NaOH. The amount of ^125^I^−^ in the collected samples of isotonic medium was determined by γ-radiation counting and expressed as fractional efflux per minute, as described previously (80). For studies of CFTR correctors, CHO cells heterologously expressing CFTR were pretreated with small molecules for 26 h at 37°C before experiments were commenced, whereas forskolin and CFTR potentiators were added to the isotonic medium bathing cells from 3 min after sample collection was initiated until the end of the experiments.

#### Patch-clamp experiments.

CFTR Cl^−^ channels were recorded in excised inside-out membrane patches using Axopatch 200A and 200B patch-clamp amplifiers and pCLAMP software (versions 6.0 and 10.3; all from Molecular Devices, San Jose, CA), as described previously (75). The pipette (extracellular) solution contained (in mM) 140 *N*-methyl-d-glucamine (NMDG), 140 aspartic acid, 5 CaCl_2_, 2 MgSO_4_, and 10 *N*-tris[hydroxymethyl]methyl-2-aminoethanesulphonic acid (TES), adjusted to pH 7.3 with Tris ([Cl^−^]; 10 mM). The bath (intracellular) solution contained (in mM) 140 NMDG, 3 MgCl_2_, 1 CsEGTA, and 10 TES, adjusted to pH 7.3 with HCl ([Cl^−^], 147 mM; free [Ca^2+^], <10^−8^ M). Using a temperature-controlled microscope stage (Brook Industries, Lake Villa, IL), the temperature of the bath solution was varied between 23 and 37°C.

After excision of inside-out membrane patches, we added the catalytic subunit of protein kinase A (PKA; 75 nM) and ATP (1 mM) to the intracellular solution within 5 min of membrane patch excision to activate CFTR Cl^−^ channels. To minimize channel rundown, we added PKA (75 nM) and ATP (1 mM) to all intracellular solutions and clamped voltage at –50 mV. The effects of temperature on the single-channel behavior of mouse CFTR were tested by increasing the temperature of the intracellular solution from 23 to 37°C in increments of 3–4°C (90). Once channel activity stabilized at the new test temperature, we acquired 4–10 min of single-channel data before increasing further the temperature and repeating the acquisition of data.

To investigate the plasma membrane stability of mouse F508del-CFTR, we monitored its thermal stability in excised inside-out membrane patches at 37°C (91). Membrane patches were excised at 27°C, and mouse F508del-CFTR Cl^−^ channels were activated by the addition of PKA (75 nM) and ATP (1 mM) to the intracellular solution. Once channels were fully activated, the temperature of the intracellular solution was increased rapidly to 37°C. To evaluate thermal stability, we calculated open probability (*P*_o_) values in 30-s intervals over a 10-min period commencing when the temperature reached 37°C (91). To test the actions of ivacaftor and genistein on mouse wild-type and F508del-CFTR, the CFTR potentiators were added to the intracellular solution in the continuous presence of ATP (1 mM) and PKA (75 nM). Because of the difficulty of removing ivacaftor from the recording chamber (91), specific interventions were compared with the preintervention control period made with the same concentrations of ATP and PKA, but without the test CFTR potentiator.

In this study, we used excised inside-out membrane patches containing up to five active channels [human wild-type CFTR, number of active channels (*n*) ≤ 5; human F508del-CFTR, *n* ≤ 5; mouse wild-type CFTR, *n* ≤ 4; mouse F508del-CFTR, *n* ≤ 3). To determine channel number, we used the maximum number of simultaneous channel openings observed during an experiment (13). To minimize errors, we used experimental conditions that robustly potentiate channel activity and verified that recordings were of sufficient length to ascertain the correct number of channels (85). Despite our precautions, we cannot exclude the possibility of unobserved F508del-CFTR Cl^−^ channels in excised membrane patches. Therefore, *P*_o_ values for F508del-CFTR might possibly be overestimated.

In most experiments, we recorded, filtered, and digitized data, as described previously (75), but in some experiments, we directly acquired single-channel data to computer hard disk after filtering at a corner frequency (*f*_c_) of 500 Hz using an eight-pole Bessel filter (model F-900C/9L8L; Frequency Devices, Ottawa, IL) and digitizing at a sampling rate of 5 kHz using a DigiData1320A interface (Molecular Devices). To measure single-channel current amplitude (*i*), Gaussian distributions were fit to current amplitude histograms, or cursor measurements were used. For *P*_o_ measurements, lists of open and closed times were generated using a half-amplitude crossing criterion for event detection and dwell time histograms constructed as previously described (75); transitions <1 ms were excluded from the analysis [8-pole Bessel filter rise time (*T*_10–90_) ∼0.73 ms at *f*_c_ = 500 Hz]. Histograms were fitted with one or more component exponential functions using the maximum likelihood method. For the purpose of illustration, single-channel records were filtered at 500 Hz and digitized at 5 kHz before file size compression by fivefold data reduction.

#### Reagents.

PKA purified from bovine heart was purchased from Calbiochem (Merck Chemicals, Nottingham, UK). Ivacaftor and lumacaftor were purchased from Selleck Chemicals (Stratech Scientific, Newmarket, UK), while genistein was from LC Laboratories (Woburn, MA) or Sigma-Aldrich (now Merck, Darmstadt, Germany). Except for NPPB-AM (88), which was a generous gift of K. L. Kirk and W. Wang (University of Alabama at Birmingham), other small molecule CFTR modulators were generous gifts of the Cystic Fibrosis Foundation CFTR Chemical Compound Distribution Program administered by R. J. Bridges (Rosalind Franklin University of Medicine and Science). They included the CFTR correctors C1 [corr-3a (57)], C2 [VRT-640 (66)], C3 [VRT-325 (84)], C4 [corr-4a (57)], C6 [corr-5c (57)], C9 [KM11060 (65)], C11 [Dynasore (49)], C12 [corr-2i (57)], C13 [corr-4c (57)], C14 [corr-4d (57)], C15 [corr-2b (57)], C16 [corr-3d (57)], and C17 [15Jf (94)] and the CFTR potentiators P1 [VRT-532 (84)], P2 [PG-01 (58)], P3 [SF-03 (58)], P4 [UC_CF_-853 (10)], P5 [ΔF508_act_-02 (93)], P7 [NS004 (33)], P8 [UC_CF_-029 (76)], P9 [UC_CF_-180 (69)], and P10 [UC_CF_-152 (69)] (https://on.cff.org/2HGbhVu). All other chemicals, including the pan-histone deacetylase (HDAC) inhibitor and proteostasis regulator suberoylanilide hydroxamic acid (SAHA; Vorinostat), were of reagent grade and supplied by Sigma-Aldrich (now Merck, Gillingham, UK).

Stock solutions of ATP were prepared in intracellular solution directly before each experiment. Forskolin was dissolved in ethanol, while CFTR modulators were solubilized in DMSO; all stock solutions were stored at –20°C. Immediately before use, stock solutions were diluted to final concentrations, and where necessary, the pH of the intracellular solution was readjusted to pH 7.3 to avoid pH-dependent changes in CFTR function (15). Precautions against light-sensitive reactions were observed when CFTR modulators were used. DMSO was without effect on CFTR activity (70, 75). On completion of experiments, the recording chamber was thoroughly cleaned before reuse (91).

#### Statistics.

**Fig. 2.**
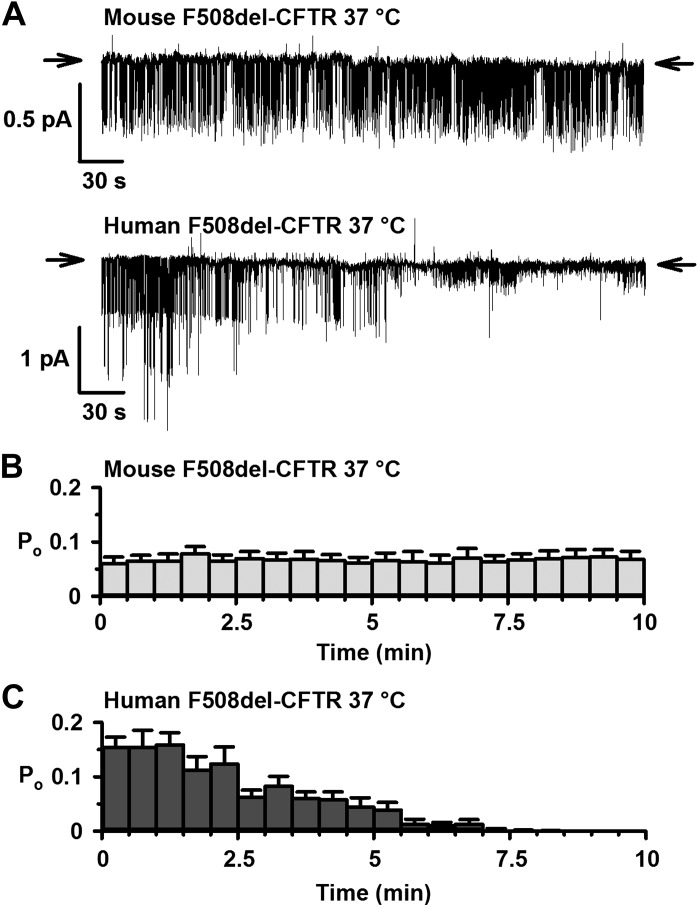
Thermostability of mouse F508del-cystic fibrosis transmembrane conductance regulator (CFTR) in excised inside-out membrane patches *A*: representative recordings of human and mouse F508del-CFTR in excised inside-out membrane patches from baby hamster kidney (BHK) and Chinese hamster ovary (CHO) cells heterologously expressing CFTR variants made in the continuous presence of ATP (1 mM) and PKA (75 nM) once channel activation was complete. Membrane patches were excised, and channels were activated at 27°C to delay temperature-dependent channel deactivation. Only after channels were fully activated was temperature increased to 37°C and thermostability evaluated. Prior to study, the plasma membrane expression of human and mouse F508del-CFTR was rescued by low-temperature incubation. Arrows denote the closed-channel state, and downward deflections correspond to channel openings. *B* and *C*: time courses of open probability (*P*_o_) for human and mouse F508del-CFTR using the conditions described in *A*. *P*_o_ values were calculated for each 30-s interval. Data are means ± SE (human F508del-CFTR heterologously expressed in BHK cells, *n* = 10; mouse F508del-CFTR heterologously expressed in CHO cells, *n* = 7). The human F508del-CFTR data in *A* and *C* were originally published in Meng et al. (51).

**Fig. 3.**
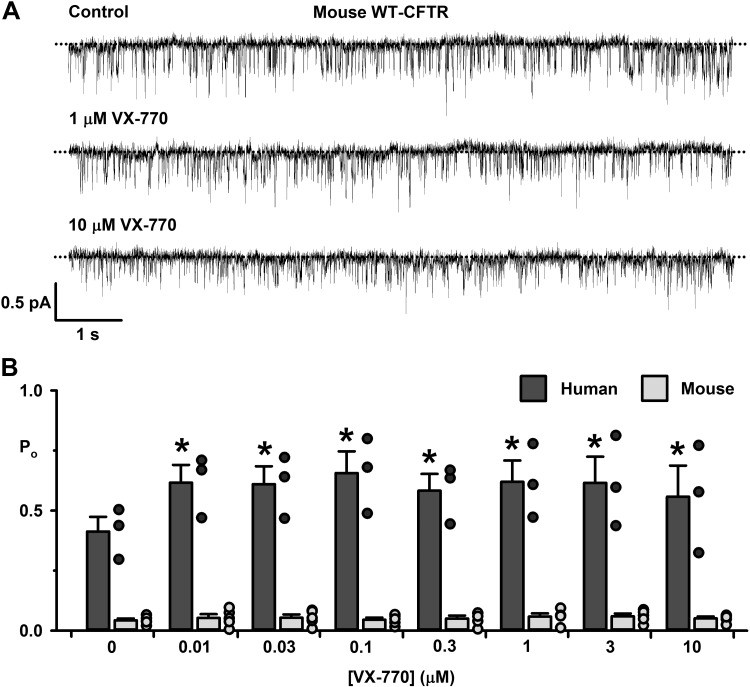
The effects of ivacaftor on the single-channel behavior of mouse wild-type cystic fibrosis transmembrane conductance regulator (CFTR). *A*: representative recordings of mouse wild-type CFTR in an excised inside-out membrane patch from a Chinese hamster ovary (CHO) cell heterologously expressing CFTR in the absence and presence of the indicated concentrations of ivacaftor added acutely to the intracellular solution. The recordings were acquired at 37°C in the continuous presence of ATP (1 mM) and PKA (75 nM) in the intracellular solution. Dotted lines indicate the closed-channel state, and downward deflections correspond to channel openings; the subconductance state of mouse CFTR is not readily apparent in these recordings. *B*: ivacaftor concentration-response relationship for human and mouse wild-type CFTR. Data are means ± SE (human wild-type CFTR heterologously expressed in NIH-3T3 cells, *n* = 3; mouse wild-type CFTR heterologously expressed in CHO cells, *n* = 5); *P < 0.05 vs. control.

**Fig. 4.**
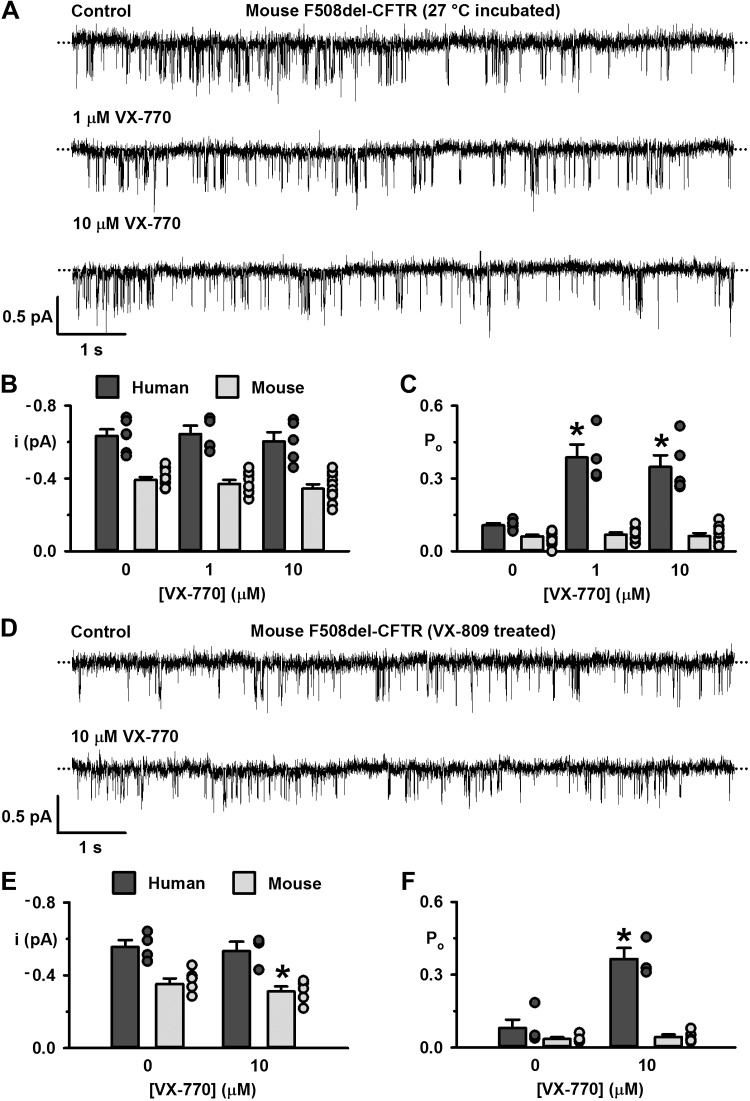
The effects of ivacaftor on the single-channel behavior of mouse F508del-cystic fibrosis transmembrane conductance regulator (CFTR) rescued by low temperature or lumacaftor. *A* and *D*: representative recordings of mouse F508del-CFTR in excised inside-out membrane patches from Chinese hamster ovary (CHO) cells heterologously expressing CFTR in the absence and presence of the indicated concentrations of ivacaftor added acutely to the intracellular solution. Prior to study, the plasma membrane expression of mouse F508del-CFTR was rescued by either low-temperature incubation (*A*) or treatment with lumacaftor (VX-809; 3 μM for 24 h at 37°C; *D*). The recordings were acquired at 37°C in the continuous presence of ATP (1 mM) and PKA (75 nM) in the intracellular solution. Dotted lines indicate the closed-channel state, and downward deflections correspond to channel openings; the subconductance state of mouse CFTR is not readily apparent in these recordings. *B*, *C*, *E*, and *F*: summary single-channel current amplitude (*i*; *B* and *E*) and open probability (*P*_o_; *C* and *F*) for the full open states of human and mouse F508del-CFTR determined from prolonged recordings (≥5 min) acquired from baby hamster kidney (BHK) and NIH-3T3 cells heterologously expressing human F508del-CFTR and CHO cells heterologously expressing mouse F508del-CFTR using the conditions described in *A* and *D* before channel deactivation (human F508del-CFTR). Dark gray and light gray circles represent individual values and columns means ± SE (*B* and *C*: human F508del-CFTR, *n* = 4–6; mouse F508del-CFTR, *n* = 8–10; *E* and *F*: human F508del-CFTR, *n* = 3–4; mouse F508del-CFTR, *n* = 5); *P < 0.05 vs. control.

**Fig. 5.**
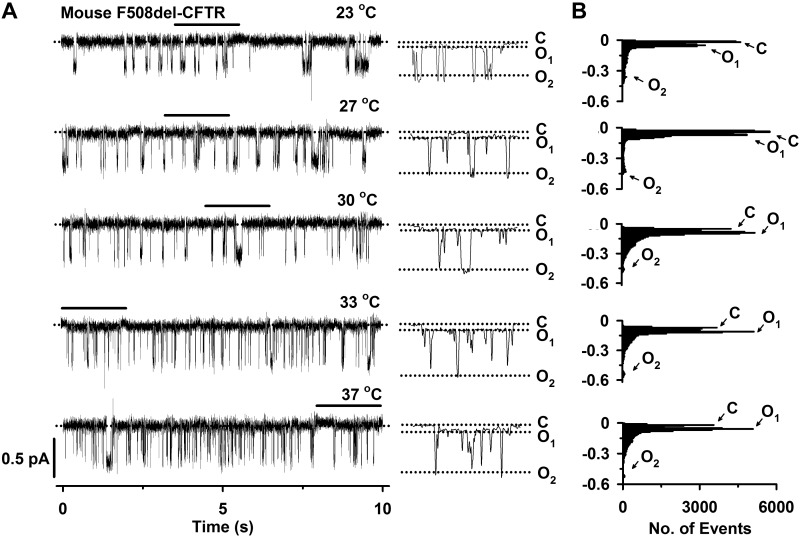
The temperature dependence of mouse F508del-cystic fibrosis transmembrane conductance regulator (CFTR) single-channel activity. *A*: representative recordings of mouse F508del-CFTR Cl^−^ channels in an excised inside-out membrane patch from a Chinese hamster ovary (CHO) cell heterologously expressing CFTR. Prior to study, the plasma membrane expression of mouse F508del-CFTR was rescued by low-temperature incubation. The recordings were acquired at the indicated temperatures in the presence of ATP (1 mM) and PKA (75 nM) in the intracellular solution. Traces on the *left* were filtered at 500 Hz, whereas the 2-s portions indicated by the bars shown on an expanded time scale to the *right* were filtered at 50 Hz. The closed-channel state (C), the subconductance state of mouse CFTR (O_1_), and the full open state (O_2_) are indicated by dotted lines. *B*: single-channel current amplitude histograms for the 10-s traces shown in *A*, filtered at 50 Hz, which removes transient events. Arrows denote C, O_1_, and O_2_.

**Fig. 6.**
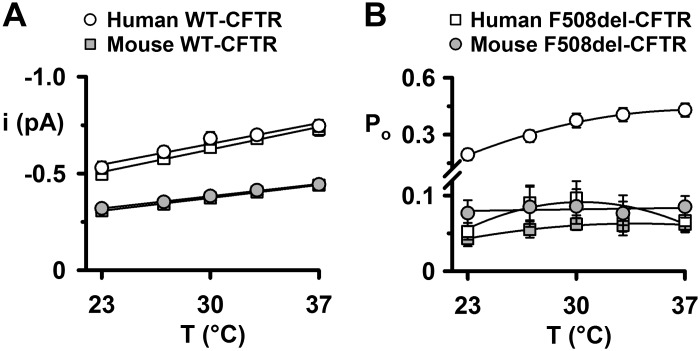
Analysis of the temperature dependence of human and mouse wild-type and F508del-cystic fibrosis transmembrane conductance regulator (CFTR) Cl^−^ channels. *A* and *B*: summary data show the change in single-channel current amplitude (*i*) and open probability (*P*_o_) between 23 and 37°C for the full open states of human and mouse wild-type and F508del-CFTR. Data are from inside-out membrane patches excised from baby hamster kidney (BHK) cells heterologously expressing human CFTR and Chinese hamster ovary (CHO) cells heterologously expressing mouse CFTR. Prior to study, human and mouse F508del-CFTR were rescued by low-temperature incubation. Data are means ± SE (human wild-type CFTR, *n* = 6–8; human F508del-CFTR, *n* = 4–8; mouse wild-type CFTR, *n* = 4; mouse F508del-CFTR, *n* = 10–12). In *A*, the continuous lines are the fit of first-order regression functions to mean data, whereas in *B*, they are the fit of second-order regression functions to mean data. Note the break in the ordinate scale in *B*. The human wild-type and F508del-CFTR data were originally published in Wang et al. (90).

**Fig. 7.**
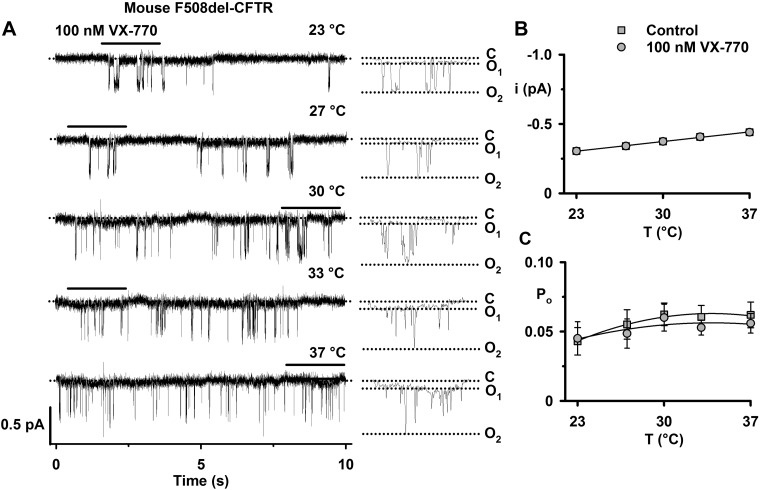
The temperature dependence of mouse F508del-cystic fibrosis transmembrane conductance regulator (CFTR) treated with nanomolar concentrations of ivacaftor. *A*: representative recordings of mouse F508del-CFTR Cl^−^ channels in an excised inside-out membrane patch from a Chinese hamster ovary (CHO) cell heterologously expressing CFTR to show the effects of acute addition of ivacaftor (VX-770; 100 nM) to the intracellular solution. The recordings were acquired at the indicated temperatures in the continuous presence of ATP (1 mM) and PKA (75 nM) in the intracellular solution. Prior to study, the plasma membrane expression of mouse F508del-CFTR was rescued by low-temperature incubation. Traces on the *left* were filtered at 500 Hz, whereas the 2-s portions indicated by the bars shown on an expanded time scale on the *right* were filtered at 50 Hz. The closed-channel state (C), the subconductance state of mouse CFTR (O_1_), and the full open state (O_2_) are indicated by dotted lines. *B* and *C*: summary data show the change in single-channel current amplitude (*i*) and open probability (*P*_o_) between 23 and 37°C for the full open state of mouse F508del-CFTR in membrane patches excised from CHO cells heterologously expressing mouse F508del-CFTR. Data are means ± SE (control, *n* = 10–12; VX-770, *n* = 5). In *B*, the continuous lines are the fit of first-order regression functions to mean data, whereas in *C*, they are the fit of second-order regression functions to mean data. In *B* and *C*, control data are the same as the mouse F508del-CFTR data in Fig. 6.

**Fig. 8.**
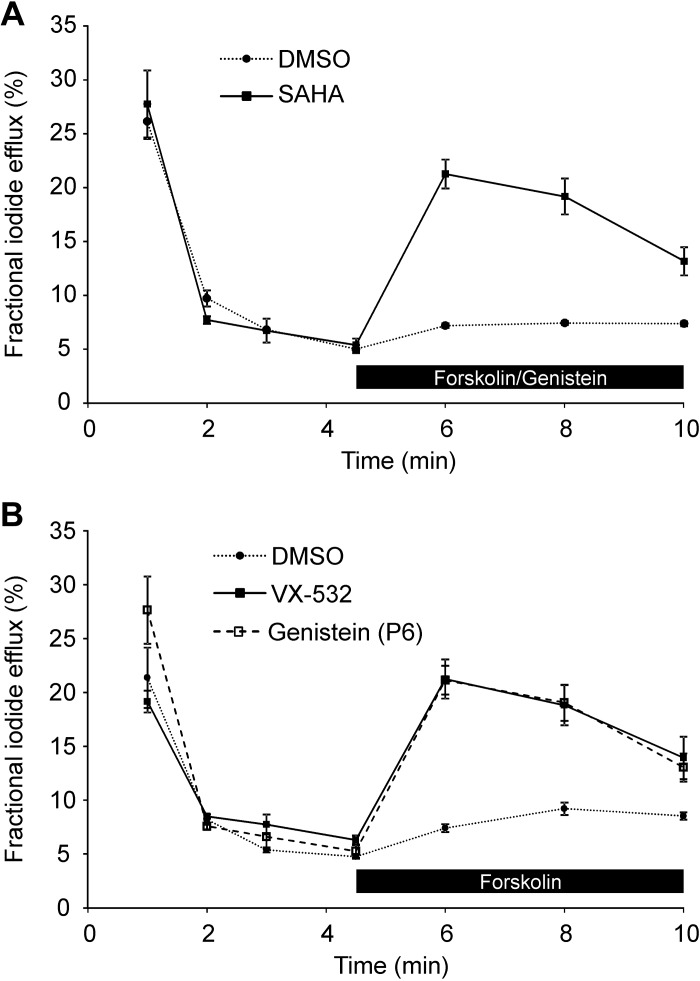
Genistein and VRT-532 potentiate iodide efflux by human F508del-cystic fibrosis transmembrane conductance regulator (CFTR) rescued by low temperature or the CFTR corrector suberoylanilide hydroxamic acid (SAHA). *A* and *B*: time courses of iodide efflux from Chinese hamster ovary (CHO) cells heterologously expressing human F508del-CFTR. In *A*, CHO cells were pretreated with DMSO (0.1% vol/vol) or SAHA (3 μM) for 26 h at 37°C before study. In *B*, CHO cells were cultured at 26°C for 26 h before study. During the periods indicated by the black bars, forskolin (10 μM) and CFTR potentiators [*A*: genistein (50 μM); *B*: DMSO (0.1% vol/vol), genistein (50 μM), or VRT-532 (10 μM)] were added to the extracellular solution. Data are means ± SE (*n* = 3).

**Fig. 9.**
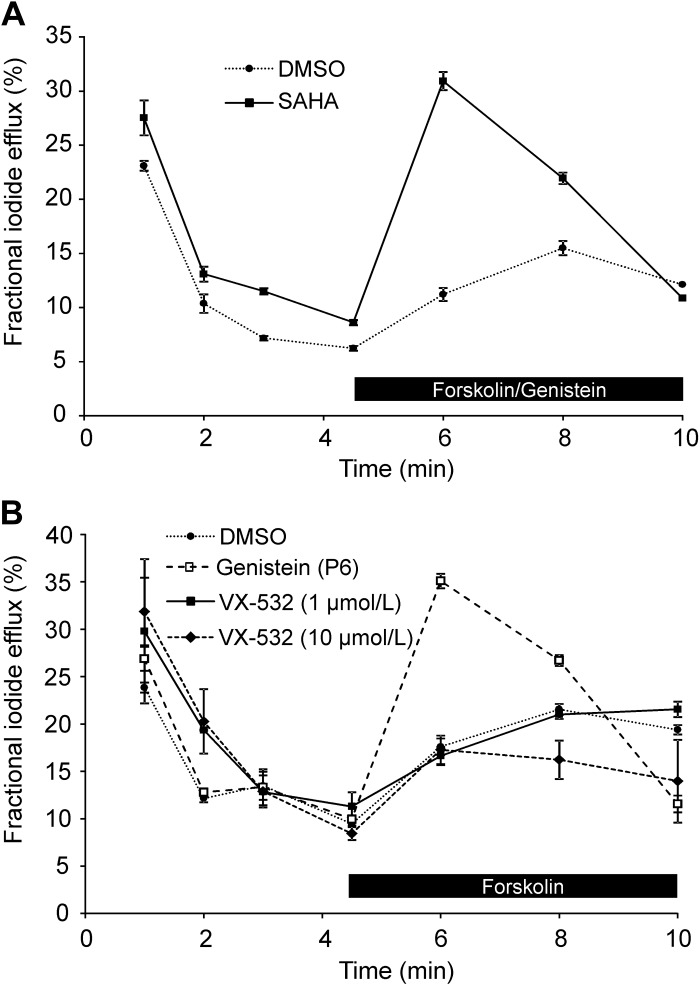
Genistein, but not VRT-532, potentiates iodide efflux by mouse F508del-cystic fibrosis transmembrane conductance regulator (CFTR) rescued by low temperature or the CFTR corrector suberoylanilide hydroxamic acid (SAHA). *A* and *B*: time courses of iodide efflux from Chinese hamster ovary (CHO) cells heterologously expressing mouse F508del-CFTR. In *A*, CHO cells were pretreated with DMSO (0.1% vol/vol) or SAHA (3 μM) for 26 h at 37°C before study. In *B*, CHO cells were cultured at 26°C for 26 h before study. During the periods indicated by the black bars, forskolin (10 μM) and CFTR potentiators [*A*: genistein (50 μM); *B*: DMSO (0.1% vol/vol), genistein (50 μM), or VRT-532 (1 or 10 μM)] were added to the extracellular solution. Data are means ± SE (*n* = 3).

**Fig. 10.**
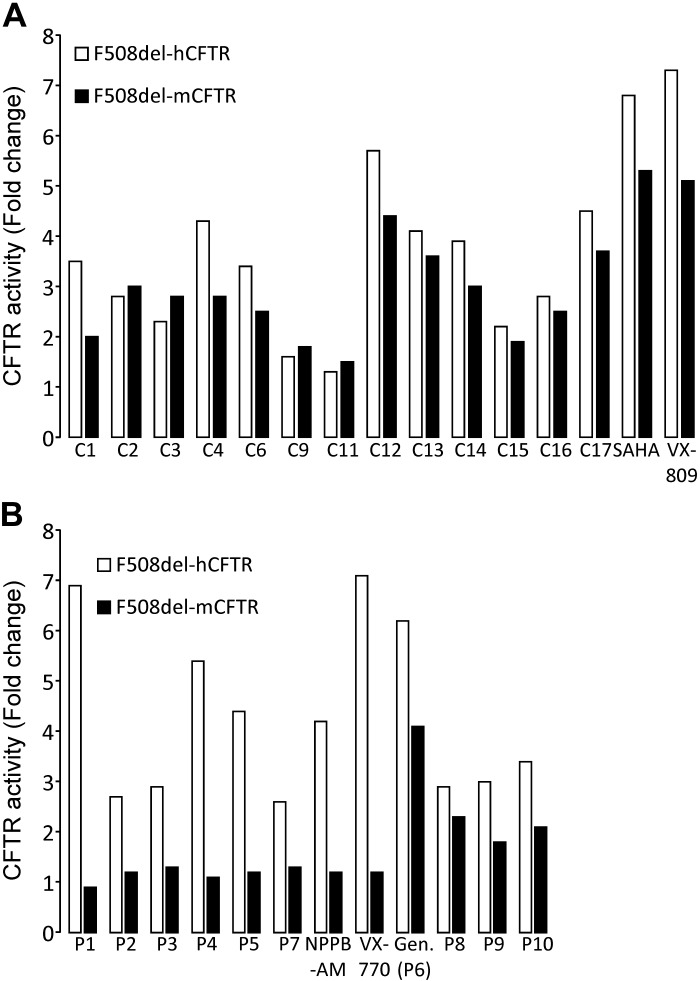
Comparison of the change in human and mouse F508del-cystic fibrosis transmembrane conductance regulator (CFTR)-mediated iodide efflux elicited by panels of CFTR correctors and potentiators. *A* and *B*: magnitude of CFTR-mediated iodide efflux in Chinese hamster ovary (CHO) cells heterologously expressing human and mouse F508del-CFTR. CFTR-mediated iodide efflux was quantified as magnitude of the initial increase in fractional ^125^I^−^ efflux at 90 s, expressed as %/min. In *A*, CHO cells were pretreated with DMSO (0.1% vol/vol) or the indicated CFTR correctors for 26 h at 37°C before study, and CFTR-mediated iodide efflux was activated by forskolin (10 μM) and potentiated by genistein (50 μM). In *B*, CHO cells were cultured at 26°C for 26 h before study and CFTR-mediated iodide efflux was activated by forskolin (10 μM) and potentiated by the indicated CFTR potentiators. The concentration of test CFTR correctors and potentiators used was optimized with concentration-response relationships over a 100-fold concentration range chosen around the most effective concentrations reported in the literature for correction and potentiation of human F508del-CFTR. Data represent mean values of two identical experiments, each performed in triplicate and expressed as fold increase in CFTR activity relative to DMSO (0.1% vol/vol) alone. For further details, see the text.

**Fig. 11.**
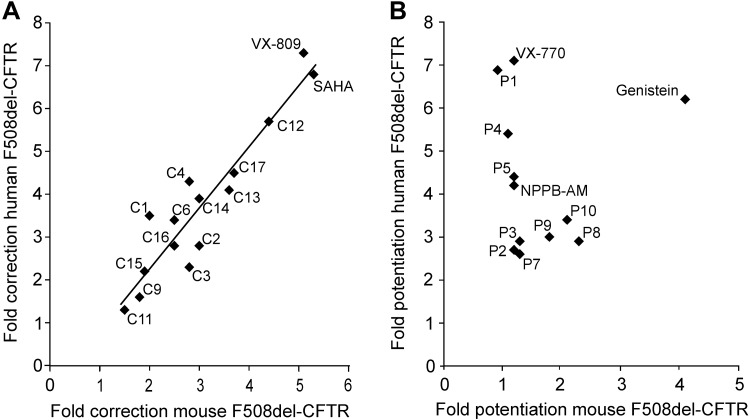
Differential responses of human and mouse F508del-cystic fibrosis transmembrane conductance regulator (CFTR)-mediated iodide efflux to CFTR potentiators, but not correctors. *A* and *B*: relationship between CFTR-mediated iodide efflux for human and mouse F508del-CFTR heterologously expressed in Chinese hamster ovary (CHO) cells and rescued by either CFTR correctors or CFTR potentiators. Data are the fold increase in CFTR activity relative to DMSO (0.1% vol/vol) alone from Fig. 10. In *A*, the continuous line is the fit of a first-order regression function to the data (*r*^2^ = 0.88). For further details, see the text.

**Fig. 12.**
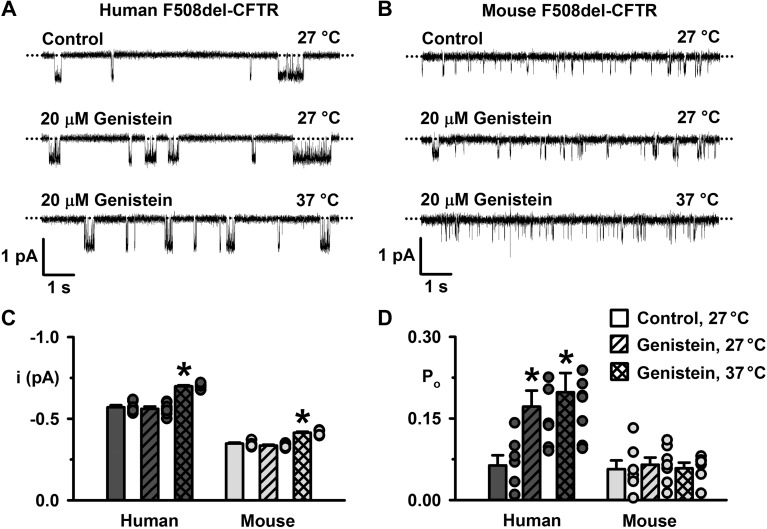
Genistein potentiates the single-channel activity of human, but not mouse, F508del-cystic fibrosis transmembrane conductance regulator (CFTR) in excised inside-out membrane patches. *A* and *B*: representative recordings of human and mouse F508del-CFTR in excised inside-out membrane patches from baby hamster kidney (BHK) and Chinese hamster ovary (CHO) cells heterologously expressing CFTR in the absence and presence of genistein (20 μM) added acutely to the intracellular solution. Prior to study, the plasma membrane expression of F508del-CFTR was rescued by low-temperature incubation. The recordings were acquired at the indicated temperatures in the continuous presence of ATP (1 mM) and PKA (75 nM) in the intracellular solution. Dotted lines indicate the closed-channel state, and downward deflections correspond to channel openings; the subconductance state of mouse CFTR is not readily apparent in these recordings. *C* and *D*: summary single-channel current amplitude (*i*) and open probability (*P*_o_) for the full open states of human and mouse CFTR determined from prolonged recordings (≥5 min) acquired from BHK cells heterologously expressing human F508del-CFTR and CHO cells heterologously expressing mouse F508del-CFTR using the conditions described in *A* and *B* before channel deactivation (human F508del-CFTR). Dark gray and light gray circles represent individual values and columns means ± SE (human F508del-CFTR, *n* = 7–8; mouse F508del-CFTR, *n* = 6–7); *P < 0.05 vs. human F508del-CFTR control.

Data recording and analyses were randomized, but not blinded. Results are expressed as means ± SE of *n* observations, but some group sizes were unequal due to technical difficulties with the acquisition of single-channel data. All data were tested for normal distribution using a Shapiro-Wilk normality test. To test for differences between two groups of data acquired within the same experiment, we used Student’s paired *t*-test. To test for differences between multiple groups of data, we used a one-way repeated-measures analysis of variance (ANOVA), followed by Dunnett’s multiple-comparison test when a statistically significant difference was observed. Tests were performed using SigmaPlot (version 13.0; Systat Software, San Jose, CA) and GraphPad Prism 5 (San Diego, CA). Differences were considered statistically significant when *P* < 0.05. In iodide efflux experiments (Figs. 8–11), *n* represents the number of monolayers of cells, whereas in patch-clamp experiments (Figs. 1–7 and 12) *n* represents the number of individual membrane patches obtained from different cells. To avoid pseudo-replication, all experiments were repeated at different times.

#### Data accessibility statement.

Data are available at the University of Bristol data repository, data.bris, at https://doi.org/10.5523/bris.1xs4o58o3va0v23ytzulm4oo76.

## RESULTS

In this study, we investigated the action of the F508del mutation on mouse CFTR. Using cell-free membrane patches from heterologous cells, we studied the single-channel behavior and thermostability of human and mouse F508del-CFTR. With the iodide efflux technique, we compared the rescue of human and mouse F508del-CFTR function by CFTR modulators. Unless otherwise indicated, all F508del-CFTR data were acquired while channel activity was stable before channel deactivation (51, 91).

### 

#### The single-channel behavior of mouse F508del-CFTR.

Figure 1 shows representative single-channel recordings and summary data of human and mouse CFTR in the absence and presence of the F508del mutation following correction of its processing defect by low-temperature incubation and channel activation by PKA-dependent phosphorylation. Consistent with previous results (46, 72), the single-channel behavior of mouse wild-type CFTR differed from human wild-type CFTR in two important respects. First, its single-channel current amplitude was reduced 40% at –50 mV (Fig. 1, *A–C*). Second, the gating pattern of mouse wild-type CFTR differed noticeably from human wild-type CFTR. For human wild-type CFTR, channel gating is characterized by bursts of channel openings to the full open state (O), interrupted by brief flickery closures and separated by longer closures between bursts; openings to subconductance states are rare for human wild-type CFTR (Fig. 1*A*). By contrast, the gating behavior of mouse wild-type CFTR is characterized by prolonged openings to a low-amplitude subconductance state (O_1_), which is not easily apparent without heavily filtering single-channel records, and many short-lived transitions to the full open state (O_2_) (Fig. 1*B*). Figure 1*D* demonstrates that the *P*_o_ of the full open state of mouse wild-type CFTR was reduced 80% compared with that of human wild-type CFTR. Because of the tiny amplitude of the O_1_ state of mouse CFTR and the paucity of excised inside-out membrane patches with only one active mouse CFTR Cl^−^ channel, we did not quantify the *i* and *P*_o_ of the O_1_ state of mouse CFTR in this study.

Prior to channel deactivation (51), the pattern of channel gating of human F508del-CFTR is characterized by infrequent channel openings separated by very prolonged channel closures (Fig. 1*A*). By contrast, the gating pattern of mouse F508del-CFTR resembled that of mouse wild-type CFTR with many brief transitions to the O_2_ state superimposed upon prolonged openings of the O_1_ state (Fig. 1*B*). Figure 1*D* demonstrates that the *P*_o_ of the O_2_ state of mouse CFTR was unaffected by the F508del mutation, whereas that of the full open state of human CFTR was decreased 80% by the mutation. Similarly, Fig. 1*C* reveals that the F508del mutation was without effect on current flow through the O_2_ state of mouse CFTR, like its effect on the *i* of human CFTR. Thus, mouse CFTR has a different gating pattern compared with human CFTR and it is unaffected by the F508del mutation.

#### Mouse F508del-CFTR exhibits thermal stability at 37°C.

One characteristic of human F508del-CFTR is reduced protein stability at the plasma membrane (48), which is evident in single-channel records as accelerated channel rundown at 37°C (71). Using excised inside-out membrane patches, we monitored the duration of F508del-CFTR channel activity at 37°C in the continuous presence of ATP and PKA by measuring *P*_o_ once channels were fully activated by PKA-dependent phosphorylation. A potential limitation of these studies is that different cells were used to investigate the plasma membrane stability of F508del-CFTR: BHK and NIH-3T3 cells for human F508del-CFTR and CHO cells for mouse F508del-CFTR. In contrast to human F508del-CFTR, which declines from *P*_o_ values of ∼0.15 to zero within 8 min, the single-channel activity of mouse F508del-CFTR was sustained; no loss of channel activity was observed over the 10-min recordings (Fig. 2). In other experiments (*n* = 5; data not shown), mouse F508del-CFTR remained fully active in excised inside-out membrane patches for ≥30 min at 37°C in the presence of PKA and ATP in the intracellular solution. This behavior of mouse F508del-CFTR differs noticeably from the thermal instability of human F508del-CFTR in excised inside-out membrane patches at 37°C (Fig. 2) (51, 91). We conclude that the F508del mutation has reduced severity on the function of mouse CFTR.

#### Differential responses of human and mouse CFTR to ivacaftor.

Some previous work using mammalian cells heterologously expressing CFTR demonstrated that mouse CFTR is unresponsive to certain agents, including ivacaftor, that potentiate human CFTR (45, 72, 82). However, experiments using *Xenopus* oocytes heterologously expressing CFTR studied at 22–23°C showed that mouse CFTR is potentiated by ivacaftor (21, 22). To investigate further the ivacaftor-sensitivity of mouse CFTR, we tested the acute effects of ivacaftor on human and mouse wild-type and F508del-CFTR using excised inside-out membrane patches. By applying the drug to the intracellular solution, we tested the direct effects of ivacaftor on the single-channel activity of CFTR at 37°C.

Figure 3*A* shows representative recordings of mouse wild-type CFTR in the absence and presence of ivacaftor (1 and 10 μM), whereas Fig. 3*B* compares the response of human and mouse wild-type CFTR to ivacaftor (10 nM to 10 μM). Visual inspection of single-channel recordings suggests that ivacaftor (1 and 10 μM) was without obvious effects on the O_2_ state of mouse wild-type CFTR (Fig. 3*A*). Consistent with this idea, there was no change in the *P*_o_ of the O_2_ state of mouse wild-type CFTR at all concentrations of ivacaftor tested (Fig. 3*B*). By contrast, ivacaftor (10 nM), the lowest concentration tested, increased the *P*_o_ of human wild-type CFTR by 49%, after which it remained elevated at all further concentrations tested (Fig. 3*B*).

Next, we tested the acute effects of ivacaftor (1 and 10 μM) on human and mouse F508del-CFTR in excised inside-out membrane patches at 37°C. For these experiments, we rescued the plasma membrane expression of F508del-CFTR by either low-temperature incubation or treatment with the CFTR corrector lumacaftor. Figure 4 shows that ivacaftor (1 and 10 μM) was without clear effect on the single-channel activity of mouse F508del-CFTR. The drug did not alter the *P*_o_ of the O_2_ state of mouse F508del-CFTR (Fig. 4, *C* and *F*). The only exception was a small, albeit significant (*P* < 0.05), reduction in the *i* of the O_2_ state of lumacaftor-rescued mouse F508del-CFTR by ivacaftor (10 μM) (Fig. 4*E*). By contrast, ivacaftor (1 and 10 μM) potentiated human F508del-CFTR rescued by either method, increasing *P*_o_ by 97–551% without altering *i* (Fig. 4, *B*, *C*, *E*, and *F*). Taken together, the data argue that under the experimental conditions tested, ivacaftor potentiates human, but not mouse, CFTR.

#### The effect of temperature on single-channel activity of mouse CFTR.

Toward an explanation for the different effects of ivacaftor on mouse CFTR observed by Cui and colleagues (21, 22) and ourselves, we reviewed the experimental conditions employed to investigate mouse CFTR. One immediate difference was temperature. Cui and colleagues (21, 22) recorded CFTR channel activity at 22–23°C, whereas we used 37°C. Therefore, we investigated the effect of temperature on the single-channel activity of mouse CFTR. Figure 5 shows representative recordings of a single mouse F508del-CFTR Cl^−^ channel at temperatures between 23 and 37°C, whereas Fig. 6 quantifies the effects of temperature on current flow through the O_2_ state and its activity for mouse wild-type and F508del-CFTR.

Visual inspection of the single-channel records in Fig. 5 revealed that temperature has marked effects on the gating behavior of mouse F508del-CFTR. At temperatures <30°C, the frequency of opening to the O_2_ state was decreased noticeably, but their duration was prolonged markedly such that most openings were to the full open state (Fig. 5*A*). By contrast, at temperatures ≥30°C, the frequency of opening to the O_2_ state was dramatically increased, but their duration was very brief, with the result that many openings appeared truncated and did not reach the amplitude of the full open state using the data acquisition conditions that we employed (Fig. 5*A*). Interestingly, openings of mouse F508del-CFTR to the O_1_ state were observed at all temperatures tested (Fig. 5*A*). However, analysis of single-channel current amplitude histograms revealed that the long closures separating openings to the O_1_ state were prolonged at temperatures <30°C, but reduced in length at temperatures ≥30°C, with the result that transitions to the O_1_ state dominated the histograms (Fig. 5*B*).

To quantify the effects of temperature on mouse CFTR, we measured the *i* and *P*_o_ of the O_2_ state. Figure 6*A* demonstrates that current flow increased 45% from 23 to 37°C for both human and mouse wild-type CFTR. Moreover, at all temperatures tested, the F508del mutation was without effect on current flow through either human or mouse CFTR Cl^−^ channels in the full open state (Fig. 6*A*). We previously demonstrated that human wild-type and F508del-CFTR exhibit strikingly different relationships between temperature and *P*_o_ (90). For human wild-type CFTR, *P*_o_ values increased progressively between 23 and 37°C, whereas for human F508del-CFTR, the relationship was bell-shaped, with a maximum *P*_o_ value of ∼30°C (Fig. 6*B*). For both mouse wild-type and F508del-CFTR, *P*_o_ values for the O_2_ state were comparable with those of human F508del-CFTR at all temperatures tested (Fig. 6*B*). Moreover, *P*_o_ values for the O_2_ state of mouse wild-type CFTR were independent of temperature, while those of the O_2_ state of mouse F508del-CFTR increased only slightly, from 23 to 37°C (Fig. 6*B*). We interpret these data to suggest that the temperature dependence of *P*_o_ for the full open state of mouse CFTR is much less than that of human CFTR.

Potentiation of human wild-type and F508del-CFTR by ivacaftor is temperature independent (90). Because Cui and colleagues (21, 22) tested the action of ivacaftor on mouse CFTR at 22–23°C, we examined the effects of ivacaftor on mouse CFTR at different temperatures. Figure 7 shows representative recordings of a single mouse F508del-CFTR Cl^−^ channel in the presence of ivacaftor (100 nM) in the intracellular solution from 23 to 37°C and summary data from five experiments. Visual inspection of the single-channel records in Figs. 5*A* and 7*A* suggest that ivacaftor (100 nM) was without effect on mouse F508del-CFTR channel gating. Neither openings to the full open state (O_2_) nor to the tiny subconductance state (O_1_) were increased. Consistent with these observations, ivacaftor (100 nM) was without effect on the* i* and *P*_o_ of the O_2_ state of mouse F508del-CFTR (Fig. 7, *B* and *C*).

#### Human and mouse F508del-CFTR have similar responses to CFTR correctors, but different responses to CFTR potentiators.

To understand better the pharmacology of mouse F508del-CFTR, we tested the effects of panels of small molecule CFTR correctors and potentiators using the iodide efflux technique. With this assay, we analyzed the behavior of a population of mouse F508del-CFTR Cl^−^ channels in intact cells. Figures 8 and 9 show time courses of iodide efflux from CHO cells heterologously expressing human and mouse F508del-CFTR, respectively. Treatment of F508del-CFTR-expressing CHO cells with the cAMP agonist forskolin (10 μM) and the CFTR potentiator genistein (50 μM) elicited robust iodide efflux from cells incubated with the CFTR corrector SAHA (3 μM for 26 h at 37°C), a HDAC inhibitor and proteostasis regulator (38), but not from cells treated with the vehicle DMSO (0.1% vol/vol) (Figs. 8*A* and 9*A*). These data suggest that SAHA rescues the plasma membrane expression of both human and mouse F508del-CFTR. They confirm and extend previous studies, which show SAHA rescue of human F508del-CFTR in CFTR-overexpressing cell models (8, 38), but not in CF and non-CF human nasal epithelial cells endogenously expressing CFTR (8) nor in small intestinal tissue from F508del CF mice (Bot AGM, Wilke M, and de Jonge HR, unpublished observations). Figure 10*A* demonstrates that 14 other CFTR correctors, including the clinically approved small molecule lumacaftor, also rescued both human and mouse F508del-CFTR. Although the efficacy of different CFTR correctors varied considerably, for any given compound, its action on human and mouse F508del-CFTR was broadly similar. As a result, there was a linear relationship between the action of CFTR correctors on human and mouse F508del-CFTR (Fig. 11*A*).

Figure 8*B* demonstrates that following low-temperature incubation, treatment of CHO cells expressing human F508del-CFTR with forskolin (10 μM) and either genistein (50 μM) or VRT-532 (P1; 10 μM) (84) mediated robust iodide efflux, whereas cells treated with forskolin and the vehicle DMSO (0.1% vol/vol) generated little or none. Figure 9*B* shows time courses of iodide efflux from CHO cells expressing mouse F508del-CFTR treated with the same agents after low-temperature incubation. Interestingly, genistein, but not VRT-532, elicited robust iodide efflux from low-temperature-rescued mouse F508del-CFTR (Fig. 9*B*). We interpret these results to suggest that genistein, but not VRT-532, potentiates mouse F508del-CFTR, whereas both agents potentiate human F508del-CFTR. When we tested 11 other CFTR potentiators on mouse F508del-CFTR, eight, including ivacaftor, were without effect, but three [UC_CF_-029 (P8), UC_CF_-180 (P9) and UC_CF_-152 (P10) (69, 76)] potentiated mouse F508del-CFTR, albeit their efficacy was noticeably less than that of genistein (Fig. 10*B*). Analysis of the effects of CFTR potentiators on human and mouse F508del-CFTR revealed that there was no relationship between the actions of compounds on human and mouse F508del-CFTR, unlike the effects of CFTR correctors (Fig. 11).

To understand better the action of genistein on mouse F508del-CFTR, we studied single channels in excised inside-out membrane patches from CHO cells expressing low-temperature-rescued mouse F508del-CFTR. As a control, we studied low-temperature-rescued human F508del-CFTR in excised inside-out membrane patches. Following the activation of human and mouse F508del-CFTR by PKA and ATP at 27°C, we added genistein (20 μM) to the intracellular solution. Once channel potentiation was complete or 5 min had elapsed, we increased temperature to 37°C and recorded channel activity for a further 5 min. Figure 12, *A* and *B*, demonstrates that genistein (20 μM) potentiated human F508del-CFTR channel gating, but was without effect on the O_2_ state of mouse F508del-CFTR. Figure 12, *C* and *D*, reveals that genistein (20 μM) was without effect on the *i* of human and mouse F508del-CFTR and the *P*_o_ of mouse F508del-CFTR, but increased the *P*_o_ of human F508del-CFTR by 170% at 27°C and by 210% at 37°C. Two independent investigators repeated this experiment using genistein (50 μM). Both found that genistein potentiated human, but not mouse, F508del-CFTR (*n* = 6; data not shown). Taken together, the data suggest that the action of genistein on mouse F508del-CFTR is mediated by a cytosolic factor, which is lost on excision of inside-out membrane patches. We conclude that CFTR correctors have similar effects on human and mouse F508del-CFTR, whereas most, but not all, CFTR potentiators enhance the activity of human, but not mouse, CFTR.

## DISCUSSION

This study investigated the single-channel function and pharmacology of mouse F508del-CFTR. In marked contrast to the mutation’s action on human CFTR, F508del was without effect on the gating behavior and thermostability of mouse CFTR. Moreover, the activity of mouse F508del-CFTR was unaffected by most of the potentiators of human F508del-CFTR tested, including ivacaftor.

Potential caveats of this study are the use of different cell lines heterologously expressing CFTR variants and no control of CFTR expression between different cells. Our rationale for using different cell lines was to optimize the acquisition of single-channel data. In previous work, we have demonstrated that the single-channel behavior of human wild-type CFTR (quantified by measuring *i* and *P*_o_) is equivalent when studied in excised membrane patches from different mammalian cells (15). The present study shows that this is also the case for human F508del-CFTR. We have not studied mouse wild-type or F508del-CFTR heterologously expressed in different cells. But comparison of our own data (Refs. 46 and 72 and the present study) with those of Ostedgaard et al. (55) suggest that the single-channel behavior of mouse wild-type and F508del-CFTR is comparable between CHO and HeLa cells. A further limitation of our study is the use of CHO cells heterologously expressing CFTR rather than airway epithelia endogenously expressing CFTR. Encouragingly, comparison of the present results with those of Cook et al. (20) demonstrate that ivacaftor has similar effects on heterologously and endogenously expressed CFTR; human, but not mouse, CFTR is potentiated by the small molecule.

The findings of the present study show some similarities, but also some differences from previous studies of mouse CFTR. In gallbladder epithelial cells, native mouse CFTR forms a PKA- and ATP-regulated Cl^−^-selective channel with a smaller single-channel conductance than that of human CFTR (32). Also consistent with the present results, the F508del mutation was without effect on the *P*_o_ of mouse CFTR (32). However, native mouse CFTR in excised membrane patches from gallbladder epithelial cells opened more frequently to the full open state than mouse CFTR heterologously expressed in CHO cells (Ref. 32 and the present results).

When heterologously expressed in mammalian cells, mouse CFTR exhibits a strikingly different pattern of channel gating compared with human CFTR, predominantly residing in a tiny subconductance state (O_1_), ∼10% of the amplitude of the full open state (O_2_), and only briefly transiting to O_2_ (Refs. 46, 55, and 72 and the present results). However, when mouse CFTR is heterologously expressed in *Xenopus* oocytes its gating pattern is characterized by stochastic transitions to two subconductance states and the full open state, with the amplitudes of the subconductance states ∼25 and ∼65% of the full open state; prolonged openings of the subconductance states are not observed, and those of the full open state are ∼66% shorter than human CFTR (22). The present results reveal that differences in the temperature used to study mouse CFTR influence its gating behavior. But this and other differences in experimental conditions [e.g., membrane voltage and extracellular Cl^−^ concentration (12)] are insufficient to explain the different behavior of mouse CFTR in excised inside-out membrane patches from mammalian cells and *Xenopus* oocytes (Refs. 22, 46, 55, and 72 and the present results). We do not attribute the difference to the mouse CFTR cDNA because Scott-Ward et al. (72) and Cui and McCarty (22) used the same supply of cDNA. One possibility is the source of PKA used to phosphorylate and activate mouse CFTR [Cui and McCarty (22), recombinant PKA; present results, PKA purified from bovine heart]. However, an alternative or additional explanation might be the different lipid compositions of mammalian cells and *Xenopus* oocytes (54). Consistent with this idea, some previous work argues that a subpopulation of CFTR molecules is recruited to cholesterol-rich membrane microdomains (lipid rafts) (1), and CFTR gating is regulated by membrane lipids (3, 61, 78). These explanations might also elucidate the different gating behavior of native and heterologously expressed mouse CFTR (Ref. 32 and the present results).

Previous work demonstrates that the severity of the F508del processing defect is species dependent. Like human F508del-CFTR (16), ferret and sheep F508del-CFTR fail to mature, whereas mouse, pig, rabbit, and shark produce some mature fully glycosylated protein (band C) and frog and chicken substantially more (2, 31, 32, 55). Less is known about the plasma membrane stability defect of F508del-CFTR across species and cell type-dependent differences in the expression of protein phosphatases, and other regulatory molecules potentially confound interpretation of results. Nevertheless, the available data suggest a gradation of severity. In excised membrane patches studied at 37°C, human F508del-CFTR heterologously expressed in BHK and HEK cells demonstrates marked thermoinstability, deactivating completely in <10 min (89, 91), whereas sheep F508del-CFTR heterologously expressed in CHO cells requires ∼15 min to deactivate completely, suggesting greater thermostability (11). Strikingly, the present results show that mouse F508del-CFTR heterologously expressed in CHO cells demonstrates thermostability at 37°C, with no loss of single-channel activity observed in excised membrane patches over a 10-min period. This behavior of mouse F508del-CFTR is reminiscent of the single-channel activity of chicken F508del-CFTR, which exhibited marked thermostability at physiological temperatures when studied in planar lipid bilayers (2).

In all species tested, the impact of the F508del mutation on channel gating is noticeably less severe than for human CFTR (reported reduction in *P*_o_: human, 80–89%; pig, 46%; chicken, 33%; sheep, 32%; and mouse, 0–50%; Refs. 2, 11, 32, and 55 and the present study). A likely explanation for the different reductions in *P*_o_ for mouse F508del-CFTR obtained by Ostedgaard et al. (55) and ourselves is the temperature used to study CFTR channel gating. Figure 6 demonstrates that at 23°C, close to the temperature used by Ostedgaard et al. (55) (25°C), the decrement in *P*_o_ is 44% compared with 27% at 37°C. Except for French et al. (32) and the present study, visual inspection of single-channel records reveals that the F508del mutation slows the rate of channel opening with the result that the interburst interval is prolonged in all species tested (2, 11, 55). These data argue that the F508del mutation disrupts the formation of the NBD1:NBD2 dimer in the ATP-driven NBD dimerization model of CFTR channel gating (86, 87). Building on these data, Jih et al. (42) demonstrated that the F508del mutation destabilized both the full and partial NBD1:NBD2 dimer configurations during CFTR channel gating. Taken together, the data suggest that structural differences between CFTR orthologs account for the spectrum of effects of the F508del mutation in CFTR processing, plasma membrane stability, and channel gating.

To explain the difference in thermostability between chicken and human F508del-CFTR, Aleksandrov et al. (2) searched for and identified the F508del-CFTR revertant mutation I539T (25, 36) and four proline residues located within dynamic regions of NBD1 (S422P, S434P, S492P, and A534P). When the I539T revertant and proline residues were introduced into the human F508del-CFTR sequence, they restored CFTR processing, thermostability, and channel gating (2), suggesting that correction of NBD1 structure is sufficient to overcome the misfolding of F508del-CFTR. However, residues S422, S492, and A534 are conserved between human and mouse CFTR, while the I539T revertant reduces the *P*_o_ of mouse F508del-CFTR (27). These data argue that other sequence changes improve the gating behavior and thermostability of mouse F508del-CFTR. Ostedgaard et al. (55) recognized that mouse CFTR has only two of the four arginine-framed tripeptides found in human CFTR, mutation of which allows F508del-CFTR to traffic to the plasma membrane (14). Besides the motifs located at R555 and R766, mouse CFTR has an additional arginine-framed tripeptide at R781 not found in human CFTR (55). Building on these data, Dong et al. (27) used human-murine CFTR chimeras to demonstrate that F508del-CFTR maturation requires NBD1, whereas rescue of F508del-CFTR channel gating necessitates NBD1-membrane-spanning domain 2 (MSD2) interactions. These results support the idea that optimal rescue of F508del-CFTR processing, stability, and function requires correction of NBD1 folding and restoration of NBD1-intracellular loop 4 (ICL4) interactions (50, 60). They highlight how the consequences of the F508del mutation are modified by the amino acid sequence of CFTR orthologs and caution that sequence changes (e.g., I539T), which overcome one defect (e.g., protein processing) might have undesirable effects on another defect (e.g., channel gating) (27).

Pedemonte et al. (59) demonstrated that cellular background influences the rescue of human F508del-CFTR by CFTR correctors, but not CFTR potentiators. By contrast, the present results reveal that in the case of human and mouse F508del-CFTR the action of CFTR correctors is unaffected by species differences, whereas that of CFTR potentiators is influenced strongly. One notable exception is the CFTR potentiator genistein, which enhanced the activity of both human and mouse F508del-CFTR in intact cells (Figs. 8*B* and 9*B*), but only human F508del-CFTR in excised inside-out membrane patches (Fig. 12). We interpret these data to suggest that the effects of genistein on mouse F508del-CFTR in intact cells is unlikely to result from its classical mode of action as a CFTR potentiator (39). Because the action of genistein on mouse F508del-CFTR is lost in cell-free membrane patches, we speculate that it acts indirectly, for example, by inhibiting the endocytosis of F508del-CFTR, possibly linked to its role as a tyrosine kinase inhibitor (47, 73).

Previous work has identified species-dependent differences in CFTR inhibition. For example, shark CFTR is insensitive to the allosteric inhibitor CFTR_inh_-172, whereas porcine CFTR is unaffected by the open-channel blocker glibenclamide (44, 75, 77). To explain these cross-species differences, Stahl et al. (77) proposed subtle differences in CFTR structure and the local environment in the vicinity of drug-binding sites. A similar explanation might account for the different effects of ivacaftor on human and mouse CFTR heterologously expressed in mammalian cells and *Xenopus* oocytes (Refs. 21, 22, and 82 and the present results). Ivacaftor accumulates in the inner leaflet of lipid bilayers disrupting lipid rafts at high concentrations (5, 17), while *Xenopus* oocytes have a distinct lipid composition to that of mammalian cells (54). However, the different effects of ivacaftor on heterologously expressed mouse CFTR in excised membrane patches and mouse models of autoimmune disease (Ref. 96 and the present results) might require a different explanation. One possibility is the action of ivacaftor on the solute carriers (SLCs) SLC26A3, SLC26A9, and SLC6A14, which modify CFTR function and hence, disease severity in CF patients (17). Another might be the different effects on mouse CFTR of acute and chronic ivacaftor treatments (Refs. 20 and 96 and the present results). Future studies should address these possibilities.

In conclusion, the F508del mutation has distinct effects on human and mouse CFTR (Table 1). The present results and other data (32, 55) demonstrate that the severity of the F508del mutation is greatly reduced in mouse CFTR. More mouse F508del-CFTR protein is delivered to the plasma membrane, where it exhibits notable thermostability and has little or no adverse effects on channel gating compared with human F508del-CFTR. The distinct effects of potentiators on human and mouse CFTR suggest that, as for pyrophosphate (72), human-mouse CFTR chimeras might be employed to investigate the interaction of these small molecules with the CFTR Cl^−^ channel. The data also suggest that CF mice have a role to play in the evaluation of small molecules that rescue the plasma membrane expression of F508del-CFTR, but not its function as a regulated Cl^−^ channel, providing an impetus for the development of humanized mouse models of CF to evaluate new therapeutics (35). Thus, subtle differences in protein structure between human and mouse CFTR strongly influence the action of the F508del mutation and the response to small molecule CFTR potentiators.

**Table 1.** Differential effects of the F508del mutation on human and mouse CFTR

	Human		Mouse	
Property	Wild-Type	F508del	Wild-Type	F508del
Protein expression	Band C	Band B	Band C	Some band C
Thermostability	Stable at 37°C	Unstable at 37°C	Stable at 37°C	Stable at 37°C
*i* (% WT human CFTR)	100	91	58	61
*P*_o_ (% WT human CFTR)	100	24	24	21
Gating pattern	Highly frequent bursts of openings	Infrequent bursts of openings	Predominantly resides in tiny subconductance state	Predominantly resides in tiny subconductance state
Effect of lumacaftor		Rescued		Rescued
Effect of ivacaftor	Potentiated	Potentiated	Insensitive	Insensitive
Effect of genistein	Potentiated	Potentiated	Potentiated	Potentiated in intact cells, but not cell-free membrane patches

Summary of effects of F508del mutation on human and mouse CFTR. For further information, see text and Ostedgaard et al. (55); *i*, single-channel current amplitude; *P*_o_, open probability; WT, wild-type.

## GRANTS

This work was funded by the Cystic Fibrosis Foundation (H. R. de Jonge) and the Cystic Fibrosis Trust (H. R. de Jonge and D. N. Sheppard). S. J. Bose was the recipient of an Industrial CASE studentship from the Medical Research Council (grant no. MR/L015919/1).

## DISCLOSURES

No conflicts of interest, financial or otherwise, are declared by the authors.

## AUTHOR CONTRIBUTIONS

H.R.d.J. and D.N.S. conceived and designed research; S.J.B., M.J.C.B., Y.W., J.L., and A.G.M.B. performed experiments; S.J.B., M.J.C.B., Y.W., J.L., Z.C., A.G.M.B., H.R.d.J., and D.N.S. analyzed data; S.J.B., M.J.C.B., Y.W., J.L., Z.C., A.G.M.B., H.R.d.J., and D.N.S. interpreted results of experiments; S.J.B., M.J.C.B., and Y.W. prepared figures; S.J.B., M.J.C.B., H.R.d.J., and D.N.S. drafted manuscript; S.J.B., M.J.C.B., H.R.d.J., and D.N.S. edited and revised manuscript; S.J.B., M.J.C.B., Y.W., J.L., Z.C., H.R.d.J., and D.N.S. approved final version of manuscript.

## ENDNOTE

At the request of the authors, readers are herein alerted to the fact that additional materials related to this article may be found at https://doi.org/10.5523/bris.1xs4o58o3va0v23ytzulm4oo76. These materials are not a part of this article and have not undergone peer review by the American Physiological Society (APS). APS and the journal editors take no responsibility for these materials, the website address, or any links to or from it.
